# Genome-wide recruitment to Polycomb-modified chromatin and activity regulation of the synovial sarcoma oncogene SYT-SSX2

**DOI:** 10.1186/1471-2164-13-189

**Published:** 2012-05-17

**Authors:** Christina B Garcia, Christian M Shaffer, Josiane E Eid

**Affiliations:** 1 Department of Cancer Biology, Vanderbilt University School of Medicine, 37232 Nashville, TN, USA; 2 Center for Human Genetics Research, Vanderbilt University, 37232, Nashville, TN, USA

## Abstract

**Background:**

SYT-SSX is the oncogene associated with synovial sarcoma (SS), a stem cell disease. SYT-SSX is thought to be responsible for sarcoma initiation and development. It interacts with components of Polycomb and SWI/SNF complexes, the two epigenetic controllers that maintain the heritable status of differentiation-specific genes in the stem/progenitor cell. Through these associations SYT-SSX is thought to alter gene expression programs by epigenetic mechanisms. Recently, we reported that SYT-SSX2 reprograms mesenchymal stem cells and myoblasts by dictating their commitment to the neural lineage while disrupting their normal differentiation. This reprogramming was due to the direct occupancy of proneural genes by the SYT-SSX2 nuclear complex. To gain a clear understanding of SYT-SSX2 control of gene expression networks, we conducted a thorough genome-wide analysis to determine the mechanism of its recruitment and identify signature sets of epigenetic markers that would predict its targeting and transcriptional activity.

**Results:**

SYT-SSX2 was recruited to distinct loci across all chromosomes, and an overwhelming number of Polycomb-modified sites enriched with the trimethylated histone H3 on lysine 27 (H3K27me3) formed the main recruiting module for SYT-SSX2. Not all SYT-SSX2/H3K27me3-occupied genes had altered expression, denoting the requirement for additional signals upon oncogene binding. Differential binding and epigenetic patterns distinguished upregulated and downregulated genes. Most activated genes had SYT-SSX2 sites enriched with H3K27me3 within their body or near their transcription start site (TSS) whereas a majority of downregulated genes were characterized by SYT-SSX2/H3K27me3-rich regions at long-range, or by modifications associated with transcription activation within the gene body or near the TSS. Hierarchical and functional clustering identified H3K27me3 as the dominant epigenetic marker associated with SYT-SSX2 binding and gene expression. Notably, this analysis revealed a cluster of upregulated neuronal genes densely covered by H3K27me3, consistent with programming toward the neural lineage by SYT-SSX2 observed previously.

**Conclusions:**

The data analysis revealed that Polycomb complexes or their modified chromatin and their stably silenced differentiation programs seem to be the main target for SYT-SSX2, suggesting that their perturbation is at the center of tumorigenesis driven by the oncogene. Further research into this mechanism is crucial to the full understanding of SS biology.

## Background

The SYT-SSX oncogene is the product of a recurrent t(X;18)(p11.2;q11.2) chromosomal translocation that characterizes synovial sarcoma (SS), a high grade, soft tissue cancer that typically arises in adolescents and young adults. Because synovial sarcoma tumors are known to develop in multiple sites of the body (with higher incidences near the joints of the lower extremities), it is believed that the disease originates in a primitive stem cell capable of differentiation into multiple lineages [1,2]. Recent studies have supported this hypothesis since human synovial sarcoma cells displayed features of multipotent stem cells whose differentiation capacity was deregulated by the oncogene [3,4], and transgenic mouse modeling revealed that tumors recapitulating SS features developed in precursors of the muscle lineage but not in more differentiated myocytes upon expression of the oncogene [5]. SYT-SSX formation is the primary event in synovial sarcoma, and its prevalent expression in the SS tumors implicates it in cancer initiation and progression [1,2]. Determining the molecular function of SYT-SSX is therefore essential for unraveling the mechanism of tumorigenesis in synovial sarcoma.

Wild type SYT (SYnovial sarcoma Translocated) and SSX (Synovial Sarcoma X) are nuclear proteins believed to function in the regulation of gene expression. SYT is required for early embryonic development [6-8] and is widely expressed in adult tissues. It lacks known DNA-binding motifs [9] and forms direct associations with key transcription modulators, including the p300 acetyl transferase (HAT; [10]), the SWI/SNF chromatin-remodeling complex ATPase, Brg1/Brm [11,12], and the repressor complex component, Sin3A [13]. All three SYT-binding proteins are known epigenetic controllers, thus these interactions are thought to regulate the coactivating function of SYT.

In contrast, the SSX genes encode a nine-member family of transcriptional corepressors located on the X chromosome whose physiological functions remain unclear [14]. Like SYT, SSX proteins lack a DNA binding domain; therefore, they may also exert their function via protein-protein interactions. It was revealed that SSX1 and SSX2 colocalize with Polycomb group (PcG) complexes through the SSXRD (SSX Repressor Domain), a highly conserved domain responsible for the bulk of its repressor activity [11,15-19]. Additional studies suggest that SSX proteins may also be targeted through direct interactions with chromatin or by binding to sequence-specific transcription factors [20,21].

When SYT-SSX forms, it retains almost the entire coding sequence of SYT, hence binding to the coactivators p300 and Brg1-SWI/SNF [11,17]. Most often, the fusion occurs with the C-terminal half of either SSX1 or SSX2 [1,2] and the association with silencing Polycomb components is preserved, creating a transcriptional imbalance: the naturally antagonistic Polycomb and SWI/SNF complexes which function in concert during development to coordinate multipotency and differentiation [22] are brought to the same genomic sites by the mutant protein. The predicted outcome is a global alteration in the nuclear programming of the SYT-SSX-expressing cell, leading to tumor initiation. Both aberrant silencing and activation of target genes via Polycomb activity manipulation occur due to SYT-SSX. SYT-SSX2 associates with the promoter of the tumor suppressor gene, EGR1, and recruits Polycomb proteins to this locus causing its repression [23]. SYT-SSX2 expression also leads to aberrant transcriptional derepression of several Polycomb targets by enhancing degradation of the Polycomb Repressive Complex 1 (PRC1) core component, Bmi1 [24]. The capacity to alter the epigenetic status of target genes also implies the persistence of the altered program in the resulting tumor. The activities of both Brg1-SWI/SNF and Polycomb maintain the expression status of genes when the initiating signal, either activation or repression, is gone [25]. This suggests that SYT-SSX could dominantly reprogram the cell through modulation of epigenetic transcriptional control.

Recently, in an effort to elucidate the initial steps of cellular transformation by SYT-SSX2, we adopted two differentiation systems, the C2C12 myoblasts and human bone marrow-derived mesenchymal stem cells (hBMMSCs). This choice was based on the desire to conduct the SYT-SSX2 studies in undifferentiated systems that were physiologically close to the two cellular backgrounds in which SS tumors developed [4,5]. In a comparative microarray analysis, we were struck by the predominance of upregulated proneural genes in both cell lines expressing SYT-SSX2 [26]. To examine how SYT-SSX2 dictated the proneural lineage, we set out to identify the specific gene loci to which the oncogene was recruited in the C2C12 myoblasts, by ChIPSeq (Chromatin Immunoprecipitation-sequencing) analysis. Correlation of SYT-SSX2 occupancy with transcription regulation within a 10Kb window from gene transcription start sites (TSS) revealed that an extensive array of neural genes was directly occupied by the oncogene in their proximal regulatory regions [26]. This result strongly suggested that SYT-SSX2 dictated commitment to the neural lineage through its physical recruitment to genes involved in diverse aspects of neural function and growth. Differentiation analysis further supported the dominant reprogramming by SYT-SSX2. Notably, while SYT-SSX2 reprogrammed the stem/progenitor cells to the neural lineage, it disrupted their normal differentiation, as the BMMSCs and C2C12 cells were unable to undergo adipogenesis and myogenesis, respectively [26].

Another significant finding was a concomitant activation by SYT-SSX2 of known controllers of stem cell growth and lineage-specification: the Wnt, fibroblast growth factor (FGF), sonic hedgehog (Shh), NOTCH, and transforming growth factor/bone morphogenetic protein (TGFβ/BMP) pathways. Notably, direct targeting and activation of the FGF receptor gene, Fgfr2, by SYT-SSX2 were responsible for the induction of the neural phenotype in the stem cells [26].

These studies uncovered a dominant function of the SYT-SSX2 oncogene in redirecting differentiation of the mesenchymal stem cells. They also revealed the activation of an autocrine signaling network that would render the stem cell autonomous and facilitate its transformation. Remarkably, the SYT-SSX2-driven effects were not only occurring in the C2C12 myoblasts and the human BMMSCs, but they were also evident in human SS tumor cells, revealing the persistent activation of SYT-SSX function throughout the life of the cancer [26].

These findings strongly suggest that aberrant reprogramming forms the basis of SYT-SSX transforming activity. To begin to understand its mechanism we decided to closely examine the nature of SYT-SSX2 recruitment to genomic loci. Recent evidence suggests that co-regulated gene subsets are characterized by common histone modification signatures. It has been shown that genes participating in similar functional pathways with identical expression patterns are marked by the same complement of histone modifications in yeast and mouse myoblasts [27,28], so combinations of markers may serve as a signature for transcriptional regulators denoting the coordinated expression of these genes. Since SYT-SSX2 interacts with epigenetic controllers, we sought to determine if a signature set of epigenetic markers associated with SYT-SSX2 occupancy exists, and to ascertain whether this specific set of markers could predict transcriptional activation or repression mediated by the oncogene. To answer these questions, we decided to conduct an in-depth analysis of the genome-wide binding of SYT-SSX2 and define the chromatin state of the genomic loci that attract the translocation.

We observed that SYT-SSX2 association with the genome is non-random, and it localizes to distinct regions. However, our most significant finding was the discovery that SYT-SSX2 occupies trimethylated histone H3 lysine 27- (H3K27me3) labeled regions within or near over 70% of positively regulated and 40% of negatively regulated genes. H3K27me3 represents the modification characteristic of Polycomb-silenced genes, and its prominent association with SYT-SSX2 supports a role for the oncogene in the re- activation of Polycomb-silenced genes. These data suggest that Polycomb complexes serve as a recruitment module for the fusion protein. An additional subset of downregulated SYT-SSX2 target genes are characterized by association of the protein with histone modifications that correlate with transcriptional activation. Taken together, there are at least 2 mechanisms of SYT-SSX2 recruitment to target genes: one dependent on PcG proteins and the other Polycomb-independent.

These results provide a fundamental basis for future research that will unravel the effect of SYT-SSX2 on the silenced differentiation programs stabilized by Polycomb and how their disruption leads to cellular transformation.

## Results

### SYT-SSX2 binding across the genome is heterogeneous and nonrandom

**Figure 1:**
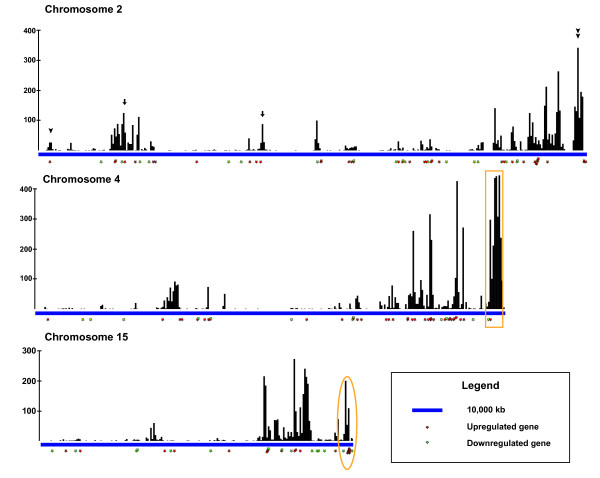
**Chromosomal distribution of SYT-SSX2 peaks.** Diagrams of chromosomes 2, 4, and 15. Chromosomes were subdivided into 500kb bins, and the number of SYT-SSX2 ChIPSeq peaks was tabulated for each bin. Black bars represent individual bins, and the height of each bar is proportional to the number of SYT-SSX2 peaks contained within that window. Scale bar shown represents a distance of 10MB. Red and green stars, respectively, depict the location of upregulated and downregulated genes determined by microarray analysis that could be annotated to SYT-SSX2 occupied regions.

As an initial step toward elucidating the mechanism of gene expression programming by SYT-SSX2 in mesenchymal precursor cells, we decided to start by examining its landing pattern in the genome. To generate a global picture of the SYT- SSX2 binding sites throughout the genome, we relied on the ChIPSeq experiment performed in C2C12 myoblasts expressing the oncogene [26]. The ChIPSeq analysis led to the identification of nearly 53,000 genomic regions (or peaks) bound by the SYT- SSX2 nuclear complex. The specificity of SYT-SSX2 peaks was confirmed by a maximum false discovery rate of 2.8% [26]. To derive the distribution pattern of SYT- SSX2 binding, we performed a sliding window analysis in which each chromosome was subdivided into 500kb bins, and the number of SYT-SSX2 peaks in each bin was tabulated. SYT-SSX2 displayed heterogeneous binding among the chromosomes as a whole and along each chromosome individually (Figure1, Additional file 1). Nearly 20% of the binding sites (9,750) are located on the X chromosome, whereas chromosome 3 has 674 binding sites (1.3%; Table1). Interestingly, areas with high levels of binding are located at chromosome ends, notably on chromosomes 2, 4, 11, 15, and X (Figure1, Additional file 1). This trend is also seen to a lesser degree on chromosomes 7, 8, 12, and 16-19 (Additional file 1). Binned binding sites appear to cluster loosely into 3 density categories: low, medium, and high. Low-density clusters are similar to the cluster centered around 5Mb on chromosome 2 and contain bins with <100 peaks (Figure1, arrowhead). Medium- density clusters contain 1-2 bins with 100-200 peaks surrounded by other bins with less than 100 peaks like the clusters centered at 28Mb or 74Mb on chromosome 2 (Figure1, arrows). The cluster centered at 179Mb on chromosome 2 (Figure1, double arrowhead) is an example of a high-density cluster which contains bins with >200 peaks with nearby bins containing >100 peaks. These data indicate that SYT-SSX2 recruitment to target loci is nonrandom and displays a preference for specific chromosomal regions.

**Table 1:** Distribution of SYT-SSX2 peaks per chromosome

**Chromosome**	**Number of ** **peaks**	**Percentage ** **of peaks**	**Chromosome**	**Number of ** **peaks**	**Percentage ** **of peaks**
1	1,651	3.1	11	3,712	7.0
2	5,311	10.0	12	2,573	4.9
3	674	1.3	13	1,145	2.2
4	6,309	11.9	14	1,493	2.8
5	4,146	7.8	15	3,202	6.0
6	1,913	3.6	16	798	1.5
7	1,846	3.5	17	1,842	3.5
8	2,759	5.2	18	836	1.6
9	1,466	2.8	19	801	1.5
10	765	1.4	X	9,750	18.4

Chromosomes were subdivided into 500kb bins, and the number of SYT-SSX2 ChIPSeq peaks was tabulated for each bin.

### SYT-SSX2 binding strongly correlates with the polycomb marker histone H3 lysine 27 trimethylation

**Figure 2:**
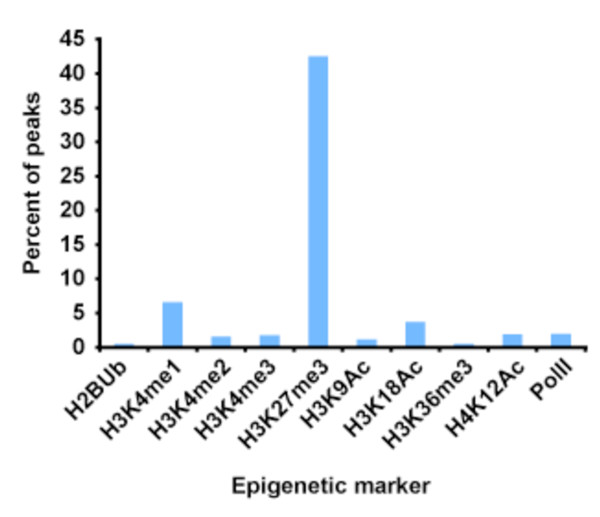
**Overlap of SYT-SSX2 peaks with epigenetic markers.** Datasets that identified regions of enrichment for histone modifications and PolII binding [28] were compared with the locations of the SYT-SSX2 peaks using the Galaxy analysis suite to determine sites of overlap. The bar graph shows the percent of SYT-SSX2 peaks that overlap with 10 epigenetic markers. Ubiquitylated histone H2 lysine 120 (H2UB) is a marker for transcription elongation. Mono, di, and trimethylated histone H3 lysine 4 (H3K4me1, H3K4me2, H3K4me3), as well as acetylated histone H3 lysine 9 (H3K9Ac), acetylated histone H3 lysine 18 (H3K18Ac), acetylated histone H4 lysine 12 (H4K12Ac), and RNA Polymerase II (PolII) all mark gene promoters and enhancers. Trimethylated histone H3 lysine 36 (H3K36me3) is a marker for active transcription in gene bodies. Trimethylated histone H3 lysine 27 (H3K27me3) is the Polycomb-specific modification associated with chromatin silencing.

After enumerating its genome-wide locations, we asked whether SYT-SSX2 is recruited to specific loci by distinct epigenetic modules. Identifying the determinants of SYT-SSX2 recruitment would reveal crucial details concerning the mechanism by which it modulates transcription. To determine if SYT-SSX2 binding might be associated with specific epigenetic markers, previously published genome-wide datasets for histone modifications and RNA polymerase II binding sites (PolII) [28] in naïve unstimulated C2C12 myoblasts were intersected with our SYT-SSX2 dataset allowing us to determine the nature of the stable epigenetic landscape to which SYT-SSX2 was recruited. Positions of histone modification enrichment and protein binding are reported as chromosomal positions, thus areas where the datasets intersect can be determined computationally. For our study, we looked for regions that overlap ≥ 1 nucleotide since SYT-SSX2 interacts with large protein complexes, and a 1-base overlap suggests close proximity to a given modification. By this method, we found quite strikingly that 22,537 SYT-SSX2-occupied regions (42.5%) overlapped with trimethylated histone H3 lysine 27 (H3K27me3; Table2, Figure2), the modification associated with Polycomb repressive complexes. This represents approximately 13% of the total H3K27me3- enriched regions in the genome of the C2C12 myoblasts (Table3), indicating that at the global level, SYT-SSX2 is preferentially targeted to a subset of Polycomb-regulated genes. Overlap with other histone modifications and PolII was not as extensive (Figure2, Tables 2 and 3). The next highest amount of overlap was seen with monomethylated histone H3 lysine 4 (H3K4me1: 3,498 peaks, 6.6%) followed by acetylated histone H3 lysine 18 (H3K18Ac: 1,961 peaks, 3.7%). This accounts for 1.3% and 0.99% of the total number of regions marked by H3K4me1 and H3K18Ac, respectively (Table3), indicating that SYT-SSX2 is associated with only a small subset of locations labeled by either of these modifications. It has been suggested that these marks identify enhancer elements [29] highlighting another possible mechanism by which SYT-SSX2 may affect gene expression.

**Table 2:** Overlap of SYT-SSX2 peaks with epigenetic markers

**Modification**	**Number of ** **peaks**	**Percent of ** **peaks**	**Modification**	**Modification Number of peaks**	**Percent of peaks**
H3K4me1	3498	6.6	H3K9Ac	595	1.12
H3K4me2	816	1.54	H3K18Ac	1961	3.70
H3K4me3	905	1.71	H3K36me3	238	0.45
H3K27me3	22,537	42.5	H4K12Ac	995	1.88
PolII	1034	1.95	H2BUb	245	0.46

The number and percent of peaks are relative to the total number of SYT-SSX2 binding sites (total = 52,992).

**Table 3:** Coverage of epigenetic markers by SYT-SSX2

**Modification**	**Number of SYT-SSX2 peaks**	**Percent of modified area**	**Modification**	**Number of SYT-SSX2 peaks**	**Percent of modified area**
H3K4me1	4,118	1.28	H3K9Ac	727	1.12
H3K4me2	955	1.32	H3K18Ac	2,210	0.99
H3K4me3	1,118	1.48	H3K36me3	275	0.17
H3K27me3	27,608	13.1	H4K12Ac	1,155	1.16
PolII	1,054	2.25	H2BUb	263	0.14

Columns 2 and 5 show the total number of SYT-SSX2 peaks overlapping with each modification. Numbers in columns 3 and 6 represent the extent to which the SYT-SSX2 peaks cover the total genomic area of each modification.

The prominence of SYT-SSX2 occupying regions enriched in H3K27me3 led to the question of their location relative to known genes. It has been shown that Polycomb complexes can mediate both short- and long-range control of gene expression [30,31], thus we determined the location of the overlapping regions between SYT-SSX2 and H3K27me3 relative to known genes. 3,692 genes could be annotated to SYT- SSX2/H3K27me3 intersecting areas, and of these, 45.6% of the peaks were located within the gene itself (Table4). Nearly 900 genes had overlapping sites from 0-5kb upstream of gene transcription start sites (TSS), and together with the genes marked by SYT-SSX2/H3K27me3 regions within the coding sequence, they account for 50% of the total SYT-SSX2-Polycomb labeled genes (Table4). These data strongly indicate that SYT-SSX2 interacts with Polycomb complexes that function at short-range. Altogether, these data illustrate that SYT-SSX2 may be preferentially targeted to specific genomic locations through interaction with Polycomb complexes and/or their associated histone modifications. This is consistent with previous studies in which SYT-SSX2 was able to associate with Polycomb proteins [23,24]. Moreover, SYT-SSX2 may function through the modulation of Polycomb activity via short-range interactions.

**Table 4:** Distribution of SYT-SSX2 peaks overlapping with H3K27me3 relative to gene transcription start sites

**Distance from TSS**	**Number of genes**	**Percent of genes**
In gene	1682	45.6
0-5 kb	872	23.6
In-5 kb	1874	50.8
0-20 kb	1672	45.3
20-50 kb	1524	41.3
>50 kb	1917	51.9

Regions of SYT-SSX2 binding intersecting with regions of H3K27me3 (≥ 1 nucleotide) were annotated to the closest TSS. A total of 3,692 genes were found to be associated with SYT-SSX2/H3K27me3 in this manner. Percent of genes refers to the number of genes with a SYT-SSX2/H3K27me3 overlapping region over 3,692.

### Differential patterns of SYT-SSX2 binding are associated with its transcriptional activity

We next inquired whether the pattern of SYT-SSX2 occupancy and its global recruitment to distinct epigenetic modifications correlated with its regulatory effects on gene transcription. We addressed this question by intersecting the ChIPSeq-derived SYT-SSX2 genomic sites with the gene profile microarray of C2C12 myoblasts expressing the oncogene [26]. By including differentially regulated genes with SYT- SSX2 binding sites at any distance upstream of the TSS or within the gene body, we identified a total of 460 upregulated and 280 downregulated genes associated with SYT-SSX2 peaks. These genes were mapped to their relative chromosomal location to determine if there was an association between the number of SYT-SSX2 peaks and gene activity. As a general trend, negatively regulated genes were associated with low- density clusters (Figure1, green dots, Additional file 1) while high-density clusters most often corresponded with positively regulated genes (Figure1, red dots, Additional file 1). Interestingly, there does not appear to be a correlation between the density of SYT- SSX2 binding and the number of genes that are either up- or downregulated. On chromosome 4, a high-density cluster is centered at 153Mb, however only 2 genes (1 upregulated and 1 downregulated) are associated with this area (Figure1, box).

Conversely, on chromosome 15, a region dense with activated genes centered at 102Mb most closely corresponds to a low-medium density cluster (Figure1, oval). Taken together, these data indicate that SYT-SSX2 binding correlates with alterations in gene activity; however, not all binding sites are associated with changes in gene expression, suggesting that SYT-SSX2 may have additional functions in the nucleus. These results are in line with a previous observation that the majority of SYT-SSX2 peaks are located at distances greater than 50kb from gene TSS [26] indicating that the oncogene may be involved in chromatin structure regulation, thereby affecting gene expression at long range.

**Figure 3:**
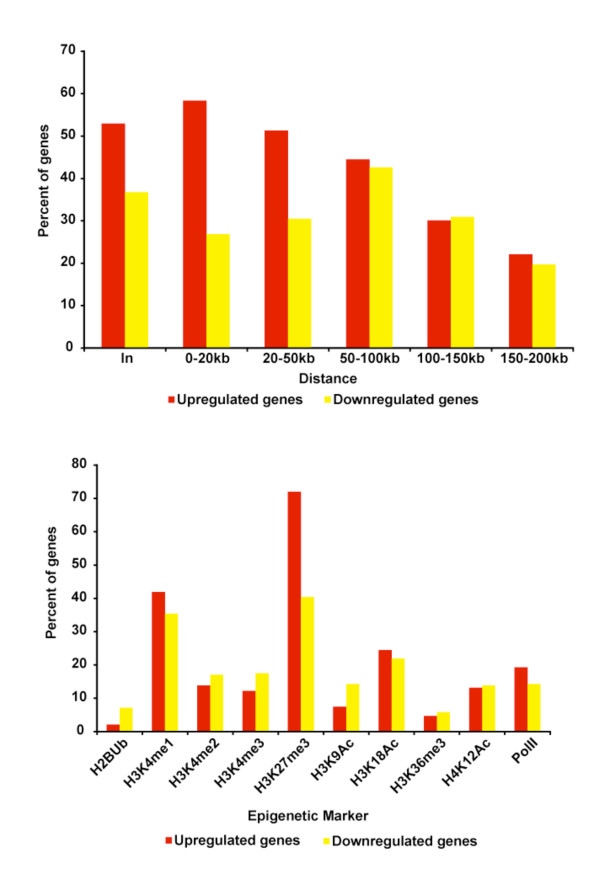
**Differential pattern of binding between upregulated and downregulated genes targeted by SYT-SSX2.** 425 upregulated and 223 downregulated genes were bound by SYT-SSX2 ranging from within the gene body up to 200Kb upstream of the TSS. Top panel shows the percentage of genes that are occupied by SYT-SSX2 within given distance windows. Bottom panel depicts a bar graph showing the percentage of genes for which the associated SYT-SSX2 occupied regions overlap with the given epigenetic markers. Red bars represent upregulated genes, and yellow bars signify downregulated genes.

We then narrowed our focus to the study of differentially regulated genes to determine whether SYT-SSX2 binding patterns could distinguish gene activation from gene repression. Interestingly, the distribution of SYT-SSX2 binding sites upstream of the TSS was markedly different depending on whether a gene was positively or negatively regulated by oncogene expression. Overall, more than half of the upregulated genes bound by SYT-SSX2 (53.9%) have at least 1 peak within a window from 0-20kb upstream of the TSS. This number decreased with increasing distance (Figure3, top panel). In contrast, 21.4% of the genes that are downregulated and bound by SYT-SSX2 have peaks within a 0-20kb window upstream of the TSS. This percentage increases with increasing distance, peaks from 50-100kb then decreases at distances between 100-150kb and 150-200kb (Figure3, top panel). These data suggest that SYT-SSX2-associated transcriptional activation is correlated with binding at close range whereas transcriptional repression associates with binding at farther distances.

### SYT-SSX2 recruitment to Polycomb-modified chromatin and enhancer elements is associated with its transcriptional activity

Recently, it has been shown that genes within specific functional groups can be distinguished by the pattern of histone modifications surrounding them. This suggests that genes within a distinct pathway have a specific epigenetic signature that allows them to be recognized by particular activating and/or repressing factors. In this way, the cell can simultaneously regulate a cohort of genes involved in a given process [27,28]. To know whether similar epigenetic markings distinguished the subsets of genes that were differentially regulated by SYT-SSX2, overlap of SYT-SSX2 peaks with histone modifications at the gene loci was determined. Seventy-two percent (72%) of the upregulated genes and 43.6% of the downregulated genes bound by SYT-SSX2 have associated peaks that overlap with H3K27me3 (Figure3, bottom panel) corroborating previous reports that the fusion protein is targeted to Polycomb-regulated genes. Surprisingly, 43.6% of the upregulated genes and 33.6% of the downregulated genes have SYT-SSX2 peaks that overlap with H3K4me1. This is significant considering that the overlap with H3K4me1 occurs with only 6.6% of the total SYT-SSX2 peaks overall. It also means that SYT-SSX2 binding to enhancer elements may be a specific feature for genes destined for altered expression. Since this modification labels enhancer elements [29], the association of SYT-SSX2 with these sites as well as Polycomb target sites suggests that SYT-SSX2 may effect transcription by modulating both enhancer and Polycomb function.

### Distinct patterns of chromatin marks characterize genes occupied and regulated by SYT-SSX2

We next decided to examine more closely the epigenetic landscape around the genes that were differentially regulated by SYT-SSX2. Our goal was to generate a model for SYT-SSX2 recruitment that would provide important leads into its mode of gene expression alteration, and determine whether distinct mechanisms are used by the fusion protein to activate or repress genes. As a first step, we tabulated the number of SYT-SSX2 peaks for each gene within a particular expression category (positively or negatively regulated) that overlapped with histone modifications, PolII binding, and DNA methylation in 5kb windows up to 50kb upstream of TSS and within the gene. We limited our analyses to this distance because of the association of SYT-SSX2 with Polycomb-marked regions at close-range relative to gene TSS and because of the difficulty in definitively assigning functional significance to binding sites at farther distances. For this analysis we also only characterized genes that had SYT-SSX2 binding sites that overlapped with at least 1 epigenetic marker and identified 314 upregulated and 110 downregulated genes by this criterion. Of these upregulated genes, 50% had overlapping sites between the fusion protein and H3K27me3 (Table5). This percentage decreases with increasing distance consistent with the trend described above with respect to all genes with SYT-SSX2/H3K27me3 intersecting regions. Also consistent with trends described above, the second most abundant overlap occurred between SYT-SSX2 and H3K4me1 within the gene body. Association between SYT- SSX2 and other histone modifications, particularly those related to transcriptional activation (but not elongation; H2UB) was also seen within gene bodies, although to a much lesser extent than either H3K27me3 or H3K4me1 (Table5). In general, the number of genes in which SYT-SSX2 associated with these other modifications decreased with increasing distance, although there are a few exceptions. Of the negatively regulated genes, the highest levels of overlap were also seen in the gene body and occurred with H3K4me1 and H3K27me3 (Table6). For H3K4me1, the number of genes with intersection of this mark with SYT-SSX2 occupancy generally decreases with increasing distance upstream of the TSS; however, from 25-30kb and 40-45kb the number of genes with SYT-SSX2/H3K4me1 regions was increased relative to the surrounding windows (Table6). For H3K27me3, a slightly different pattern is seen. The number of genes with SYT-SSX2/H3K27me3 sites decreases dramatically from 0- 5kb but increases with increasing distance upstream of the TSS (Table6). With downregulated genes, SYT-SSX2 also appears to associate with modifications related to active transcription. Regions enriched in H3K4me2, H3K4me3, H3K9Ac, H3K18Ac, H4K12Ac, and PolII occupancy overlap with SYT-SSX2 binding sites in over 10% of the downregulated genes, and markers associated with transcriptional elongation (H3K36me3 and H2BUb) overlap with SYT-SSX2 sites in more than 5% of the downregulated genes (compared with less than 3% of the upregulated genes). In summary, SYT-SSX2 associates with epigenetic markers, particularly H3K27me3 and H3K4me1. Most of the upregulated genes in this analysis are marked by H3K27me3, and SYT-SSX2 appears to bind close to the TSS. In contrast, SYT-SSX2 occupies H3K4me1- or H3K27me3-enriched regions in a similar percentage of downregulated genes and also associates with more markers of transcriptional activation and elongation.

**Table 5:** Distribution of SYT-SSX2-overlapping epigenetic markers relative to transcription start sites of upregulated genes

**Marker**	**In**	**0-5**	**5-10**	**10-15**	**15-20**	**20-25**	**25-30**	**30-35**	**35-40**	**40-45**	**45-50**
DNA me	9.55	4.46	0.32	0.96	0.32	0	0	0.64	0.32	0.64	0.64
H3K4me1	18.8	5.10	2.23	5.41	5.73	2.55	6.37	3.82	4.46	4.46	5.73
H3K4me2	8.28	5.10	0.32	1.27	0.64	0.64	0.96	0.32	0.96	0.96	0.64
H3K4me3	8.60	4.14	0.64	1.27	0.32	0.64	0.64	0.32	0.64	1.27	0.96
H3K27me3	50.0	31.2	29.3	25.7	23.9	24.5	23.6	21.3	21.3	22.0	19.4
H3K9Ac	5.10	0.64	0.32	0.64	0	0.32	0.96	0.32	0.64	1.91	0.64
H3K18Ac	9.24	2.23	1.91	2.23	2.55	2.55	2.55	2.23	3.82	4.78	3.50
H3K36me3	2.87	0	0	0.32	0.64	0.32	0.32	0.96	0	0.96	0.32
H4K12Ac	7.32	1.27	0.64	1.59	1.27	0.96	0.64	1.27	1.59	1.91	0.96
H2BUb	1.27	0	0	0.32	0.32	0.64	0.32	0.64	0	0.32	0
PolII	10.2	3.18	1.59	1.91	1.27	1.27	1.27	1.59	1.27	2.23	2.55

Percentages are relative to the total number of upregulated genes with SYT-SSX2 peaks that overlap any epigenetic marker from 0-50kb upstream of the TSS and including the gene body (total = 314). Numbers in first row represent distances measured in kilobases.

**Table 6:** Distribution of SYT-SSX2-overlapping epigenetic markers relative to transcription start sites of downregulated genes

**Marker**	**In**	**0-5**	**5-10**	**10-15**	**15-20**	**20-25**	**25-30**	**30-35**	**35-40**	**40-45**	**45-50**
DNA me	7.27	4.55	0.91	0.91	0	0.91	0.91	0	0	0.91	2.73
H3K4me1	23.6	8.18	9.09	8.18	5.45	3.64	8.18	5.45	3.64	8.18	1.82
H3K4me2	17.3	10.0	3.64	1.82	1.82	3.64	3.64	0	0	4.55	2.73
H3K4me3	17.3	10.9	2.73	3.64	1.82	4.55	2.73	0.91	0.91	3.64	6.64
H3K27me3	22.7	3.64	10.0	7.27	7.27	7.27	12.7	8.18	11.8	15.5	16.4
H3K9Ac	14.6	9.09	4.55	3.64	2.73	1.82	1.82	0	0	2.73	1.82
H3K18Ac	17.3	3.64	7.27	6.36	4.55	2.73	3.64	0	4.55	3.64	3.64
H3K36me3	8.18	0.91	1.82	1.82	0.91	2.73	0.91	0.91	1.82	0	0
H4K12Ac	12.7	9.09	3.64	7.27	1.82	3.64	1.82	0	0	3.64	2.73
H2BUb	7.27	0	1.82	0.91	0	1.82	0	0.91	1.82	0	0.91
PolII	18.2	10.0	3.64	0.91	4.55	2.73	1.82	2.73	0.91	2.73	4.55

Percentages are relative to the total number of downregulated genes with SYT-SSX2 peaks that overlap any epigenetic marker from 0-50kb upstream of the TSS and including the gene body (total = 110). Numbers in first row represent distances measured in kilobases.

### Hierarchical and functional clustering of differentially regulated genes by SYT- SSX2

In order to determine higher order relationships among the histone modifications themselves and gene expression, and using the criterion that genes were included if they contained a binding site for SYT-SSX2 that overlapped with at least 1 epigenetic marker, hierarchical clustering was performed on the differentially regulated genes. To do so, the degree of overlap between SYT-SSX2 and a given modification was calculated as a ratio of bases covered per 5kb bin upstream of the TSS or the ratio of bases covered in the gene body over the total number of bases in the coding sequence. This data generated a signature of modifications by distance for each gene and was used in hierarchical clustering analyses.

**Figure 4:**
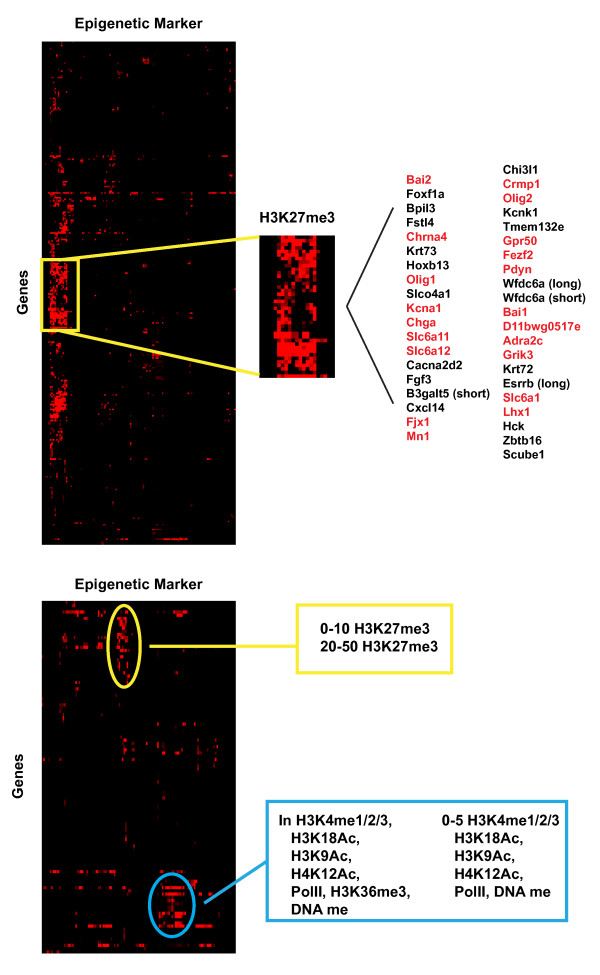
**Hierarchical clustering of differentially regulated genes.** Signatures for each differentially regulated gene were derived from the extent of overlap between SYT-SSX2 peaks and the epigenetic markers within the gene body or in 5Kb bins upstream of the TSS up to -50Kb (defined as coverage ratio; each bin has a coverage ratio for each epigenetic marker). Signatures were used as the input for hierarchical clustering using Cluster 3.0. Heatmaps were generated using Java Treeview. Each row represents a gene, and each column represents an epigenetic marker for a given bin (for example H3K27me3 from 0-5Kb or PolII in gene). Coverage ratios range from 0-1 with black squares representing bins with a ratio of 0 and red squares representing a ratio of 1. Top panel: Clustering of upregulated genes. Inset is enlarged portion of the heat map, and the epigenetic markers included in this region correspond to H3K27me3 at all distances and within the gene. Gene names are listed to the right. Highlighted in red are genes that are involved in neural development and function. Bottom panel: Clustering of downregulated genes. Yellow and blue ovals isolate 2 distinct clusters corresponding to genes with SYT-SSX2/H3K27me3 overlapping regions and SYT-SSX2 peaks overlapping with modifications associated with transcriptional activation, respectively. The position and identity of the epigenetic marker(s) comprising a particular cluster are listed to the right.

Analysis of the upregulated genes corroborated earlier results and identified H3K27me3 as the predominant modification associated with SYT-SSX2 binding and gene expression (Figure4, top panel). The location and extent of H3K27me3 was variable across all genes, but there were 2 sub-clusters in which SYT-SSX2/H3K27me3 intersecting sites were located within the entire range of distances that we analyzed. The first of those sub-clusters is highlighted in Figure4 (top panel). It has been reported previously that genes densely covered by H3K27me3 were involved in the differentiation and development of alternate lineage pathways, thus we wanted to determine the function of the genes within this sub-cluster. Based on our previous analysis [26], we found that 50% of these genes are involved in neural development and function. To summarize, SYT-SSX2 occupies regions within and upstream of upregulated genes that are enriched in H3K27me3. Functionally, these genes can be subdivided based on the extent of SYT-SSX2/H3K27me3 intersection and are in line with our previous observation of the increased expression of genes with neural characteristics.

Similar hierarchical clustering was performed on the downregulated genes. This analysis led to the identification of 2 clusters of genes with differential signatures. The first is characterized by SYT-SSX2/H3K27me3 overlap from 0-10kb and 20-50kb upstream of gene TSS, whereas the second cluster is marked by the overlap of SYT- SSX2 with histone modifications related to transcriptional activation at close ranges (Figure4, bottom panel). Interestingly, these two signatures appear to be mutually exclusive. SYT-SSX2/H3K27me3 overlaps are minimal or absent in the genes marked by close-range SYT-SSX2 intersection with activating modifications and vice versa (Figure4, bottom panel). Additionally, unlike the upregulated genes, which were functionally related based on their clustering, the genes within these clusters were not clearly associated with a particular pathway or program. Together, these data suggest that SYT-SSX2-mediated downregulation of gene expression occurs through different mechanisms, one dependent on recruitment by Polycomb and the other independent of Polycomb.

## Discussion

In this report we present evidence that the nuclear recruitment of the SYT-SSX2 oncogene is a non-random event and that it is directed to specific loci across the genome. Polycomb-modified regions form the principal targeting module for the SYT- SSX2 nuclear complex, supporting earlier observations regarding the association of the oncogene with Polycomb components [11,17,24]. In addition, a subset of the genes bound by SYT-SSX2 displayed alterations in expression. These genes were typified by certain epigenetic attributes: upregulated genes were characterized by the predominant association of SYT-SSX2 with regions enriched with the Polycomb marker H3K27me3, whereas downregulated genes could be subdivided into at least 2 categories distinguished by occupation of the fusion protein in regions displaying either H3K27me3 enrichment at short- and long-ranges or the presence of modifications associated with transcriptional activation within the gene body or near the TSS.

Our genome-wide analyses revealed that SYT-SSX2 is targeted to over 3,000 genes, yet alterations in expression are noted for, at most, 740 of these targets. It is not unprecedented that the number of binding sites for a particular factor is far greater than the number of genes that are differentially expressed when that factor is induced, as was the case with the myogenesis regulator MyoD [32], or the PAX3-FKHR fusion associated with rhabdomyosarcoma [33]. These data also suggest that additional signals may be required in order to produce functional outcomes after binding to target loci. In support of this notion, phosphorylation of bound p53 upon treatment with etoposide was required to alter gene expression [34]. In the same way, alterations in gene expression by SYT-SSX2 may require additional signaling events that succeed its binding. The H3K27me3 modification is catalyzed by PRC2, and it is known that PRC1 and PRC2 do not occupy completely identical sets of genes within a given cell type [28,35]. As direct interaction of the fusion protein has only been seen with the PRC1 component Ring1b [24], it follows that SYT-SSX2 will not associate with all H3K27me3-labeled regions. Furthermore, PRC1 may also be recruited to chromatin independently of PRC2 [25]. Recruitment of SYT-SSX2 by PRC1 could then explain at least some of the other binding sites that are not enriched for H3K27me3. Therefore, it would be interesting to determine the degree of overlap between SYT-SSX2 ChIPSeq and genome-wide binding patterns of PRC1 in C2C12 cells.

Additional signatures distinguished upregulated and downregulated genes. Most genes with increased expression and marked by H3K27me3 had SYT-SSX2 binding sites within their body or near their TSS, while greater numbers of genes with decreased expression marked by H3K27me3 are occupied at a distance. This dissimilarity may reflect alternate mechanisms of Polycomb-mediated silencing. For example, it has been reported that the structure of PRC1 may differ when it is proximal to the TSS versus when it is bound distally; functionally, this results in opposite consequences on gene expression after depletion of PRC1 components [36]. Therefore, PRC1 dysfunction caused by SYT-SSX2 could result in opposite effects. Another explanation may involve the ability of SYT-SSX2 to interact with Brg1. In embryonic stem (ES) cells, Brg1 tunes expression of Polycomb target genes resulting in either activation or enhanced silencing, and so may augment repression rather than antagonize it [37].

In addition, a second subcluster of downregulated genes was characterized by the presence of histone modifications associated with active transcription within the gene or proximal (0-5kb) to the TSS. Together with the fact that close-range binding by SYT-SSX2 at Polycomb-regulated genes results in gene activation, these data indicate that proximal binding by the fusion protein functions to antagonize the transcriptional status of target genes. Moreover, previous work has indicated that these are both consequences of aberrant Polycomb function since SYT-SSX2 has been shown both to antagonize and to initiate Polycomb silencing [23,24]. In this way, SYT-SSX2 may act as a switch protein that generally opposes the gene expression profile of the cell.

Clustering of neuronal genes occupied by SYT-SSX2 and enriched with H3K27me3-marked chromatin provided a link to the mechanism of proneural lineage activation by the oncogene [26]. More specifically, it begins to explain how dictation of the neuronal program at the expense of commitment to the normal mesenchymal lineages might have taken place. In a recent report on the epigenetic landscape during myogenic differentiation of the C2C12 myoblasts, gene subsets pertaining to permanently silenced programs including neurogenesis appeared to be held in check by an abundance of modified histone H3K27me3, reflecting stable silencing by Polycomb [28]. They represent non-mesodermal lineages whose activation during myogenesis would be inappropriate and disruptive to the myoblast. Intriguingly, these programs are believed to be distinctly regulated by PRC1, and are not influenced by PRC2 [28]. SYT-SSX2 has so far been shown to specifically deregulate the silencing function of the Bmi/Ring1 E3-ligase of PRC1 [24]. It follows that SYT-SSX2 may be recruited to H3K27me3 sites by PRC1, resulting in the untimely activation of PRC1-controlled lineages such as neurogenesis. Further investigations into the mechanism of PRC1 antagonism upon recruitment of SYT-SSX2 and the factors involved are needed to clarify how the neuronal lineage is activated and the extent of its contribution to normal differentiation block and initiation of tumorigenesis.

Additional mechanisms can explain the specificity for the upregulation of neural genes. The first involves the endogenous expression of certain factors that makes a particular outcome more likely in one cell versus a different type. It is hypothesized that expression is the result of the balance between Polycomb and Trithorax activity at a given gene, and some cell types may possess additional regulators that can affect gene expression once that balance has been perturbed [38,39]. C2C12 cells may then express certain factors that could guide the expression of neural genes. One potential factor is the REST/NRSF transcriptional repressor that silences neural genes in alternate lineages. Inhibition of its activity leads to neurogenesis in C2C12 cells so misexpression of its target genes by SYT-SSX2 may result in the ectopic neural program seen in these cells [40]. In a precedent example, SYT-SSX was reported to cause aberrant expression of E-cadherin by interacting with either of the tissue-specific transcriptional repressors Snail or Slug [41]. This suggested that interaction with repressor molecules directs gene activation by the fusion.

An alternate mechanism may involve the activation of tissue-specific enhancer elements. In ES cells, enhancers that control the expression of inactive genes involved in differentiation of multiple lineages are labeled by H3K27me3 and H3K4me1. When these elements become active K27 becomes acetylated, a modification that can be catalyzed by p300 [42,43]. Recruitment of SYT-SSX2 to these elements by interactions with Polycomb may lead to increased acetylation of K27 by p300 resulting in their activation and subsequent perturbations in the balance between silencing and expression.

**Figure 5:**
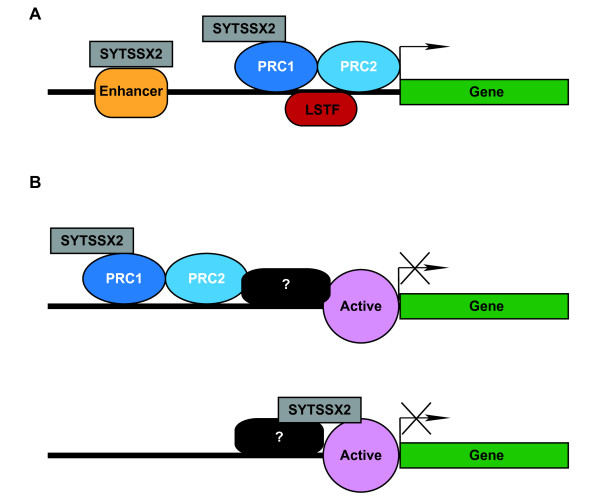
**Models of SYT-SSX2 recruitment and activity. A**)Model of recruitment and activity at an upregulated gene. SYT-SSX2 is recruited primarily to silent genes involved in the specification of other lineages by interactions with Polycomb Repressive Complexes (PRC1, dark blue oval; PRC2, light blue oval). In the presence of certain lineage-specific transcription factors (LSTF, red oval) and/or by interaction with lineage-specific enhancers (Enhancer, orange rounded rectangle), SYT-SSX2 association with the gene results in transcriptional activation. **B**) Model of recruitment and activity at a downregulated gene. Top) SYT-SSX2 may be recruited by interactions with Polycomb Repressive Complexes at a distance from the gene TSS. The gene itself is marked by activating histone modifications (Active, pink circle), and due to the presence of other regulators (?, black rounded rectangle), SYT-SSX2 carries out transcriptional silencing. Bottom) SYT-SSX2 may be directly recruited to the proximal regulatory region through association with activating histone modifications or other unidentified factors. These other factors also provide a signal to SYT-SSX2 such that it mediates repression.

These data allow us to propose a model of recruitment and regulation of target gene expression by SYT-SSX2. In the case of upregulated genes, the fusion protein is recruited by interactions with PRC1, and gene activation is determined by the presence of lineage-specific transcription factors or the activation of specific enhancer elements (Figure5A). For downregulated genes, SYT-SSX2 may be recruited by PRC1 or PRC2 at a distance from target promoters (Figure5B, top) or directly targeted to active genes (Figure5B, bottom). Recruitment may occur through interactions with the modified histones themselves, the complexes that catalyze those modifications, or additional proteins like sequence-specific transcription factors. The presence of other factors is likely to be important in specifying target genes for repression.

## Conclusions

The data described here provide a foundation for uncovering the mechanism of SYT-SSX2 recruitment. The preeminent association of SYT-SSX2 with H3K27me3 indicates that the fusion protein does not simply target to regions of open chromatin by default, but it occupies a subset of Polycomb loci. Most importantly, this analysis has revealed that nuclear programs stably silenced by Polycomb complexes in any mesenchymal precursor cell could potentially constitute the primary target for deregulation by SYT-SSX2. It provides a justification for the apparent dominant effect of the oncogene in its target cells and the ability of synovial sarcoma to arise in multiple sites of the body.

As binding of SYT-SSX2 does not completely correlate with changes in gene expression, additional targeting factors, genetic or epigenetic, as well as specific extracellular signals are most likely necessary to provide the proper stimulus for activation of distinct gene subsets and the generation of distinct cell fates. Thus, genes that are bound by SYT-SSX2 but whose expression is unaltered may be poised for activation in response to certain signaling events. Indeed, the dependence of the neural phenotype on FGF signaling supports such a scenario [26].

This analysis sets the stage for key developments toward the full understanding of how SYT-SSX2 redirects a heritable fate in the undifferentiated stem cell and identifying the factors and the extrinsic and intrinsic signals involved in this process. The Polycomb proteins are important regulators of gene expression in development as well as cancer, and much attention has focused on the mechanism through which these proteins regulate differentiation and contribute to tumorigenesis. One aspect of Polycomb function that has not been addressed extensively is how the activity of these proteins is regulated. As deregulation of Polycomb activity seems to be at the heart of SYT-SSX2 function, the differential binding patterns and their correlation with gene expression described in this report provide a unique opportunity to examine this process in detail.

Moreover, as the fusion protein demonstrated a propensity to target the stem cell signaling network [35], the manner in which these pathways regulate Polycomb function can also be explored. Elaborating how SYT-SSX2 targets and controls a specific program will clarify how to change the epigenetic structure of one cell to that of an alternate lineage. Ultimately, this will improve the efficiency of cellular reprogramming, and more importantly, is essential to understanding the biology of synovial sarcoma.

## Methods

### Data accessibility

All microarray and ChIPSeq data are available at the Gene Expression Omnibus (available at http://www.ncbi.nlm.nih.gov/geo/; [44]) accessions GSE26562 (C2C12 microarray), GSE26563 (hMSC microarray, GSE26564 (SYT-SSX2 ChIPSeq), and GSE26565 (accession for all datasets).

Previously published ChIPSeq datasets for ubiquitylated histone H2B (H2BUb); mono-, di-, and trimethylated histone H3 lysine 4 (H3K4me1/2/3); acetylated histone H3 lysine 9 (H3K9Ac), lysine 18 (H3K18Ac), and histone H4 lysine 12 (H4K12Ac); trimethylated histone H3 lysine 27 (H3K27me3) and lysine 36 (H3K36me3); and RNA polymerase II (PolII) were downloaded from the Gene Expression Omnibus (GEO) accession GSE25308 [28]. MeDIP-ChIP data were obtained from GEO accession GSE22077 [45]. Overlapping regions between individual datasets and SYT-SSX2 were determined using the Coverage and Intersect tools from the Galaxy program (available at http://main.g2.bx.psu.edu/) [46-48].

### Association of SYT-SSX2 ChIPSeq peaks with gene expression

SYT-SSX2 peaks were annotated to the nearest downstream gene on both strands by measuring the distance from the 5’ end of the peak to the gene transcription start site (TSS). Peaks associated with differentially expressed genes were identified.

### Hierarchical clustering

Differentially regulated genes containing overlapping sites between SYT-SSX2 and specific epigenetic markers within the gene and up to 50kb upstream of the TSS were used in the clustering analysis. For each gene, coverage ratios (the number of bases covered by overlapping regions divided by the total number of bases in a given window) for the gene body and for 5kb bins upstream of the TSS (up to 50kb) were calculated for each epigenetic marker. These coverage ratios served as the input data for the clustering analysis. Hierarchical clustering was performed separately for up- and downregulated genes using Cluster 3.0 [49] with gene and array clustering. The similarity metric used was Spearman Rank Correlation, and the clustering method used was centroid linkage. The output file was uploaded to Java Treeview [50] for visualization. Heat map images were downloaded from Java Treeview.

## Competing interests

The authors declare that they have no competing interests.

## Authors’ contributions

CBG, CMS, and JEE all collaborated on performing the analyses and producing results. CBG and JEE planned the project and wrote the manuscript. All authors read and approved the final manuscript.

## Supplementary Material

Additional file 1 Genomic Distribution of SYT-SSX2 Binding Sites. Click here for file
