# Supplementary material for: Genome-wide recruitment to Polycomb-modified chromatin and activity regulation of the synovial sarcoma oncogene SYT-SSX2
Source: BMC Genomics. 2012 May 17;13:189. doi: 10.1186/1471-2164-13-189 (PMC3460777; doi:10.1186/1471-2164-13-189)
Supplement: Additional file 1 — Genomic Distribution of SYT-SSX2 Binding Sites. [file 1471-2164-13-189-S1.pdf]

Additional File 1: Genomic Distribution of SYT-SSX2 Binding Sites

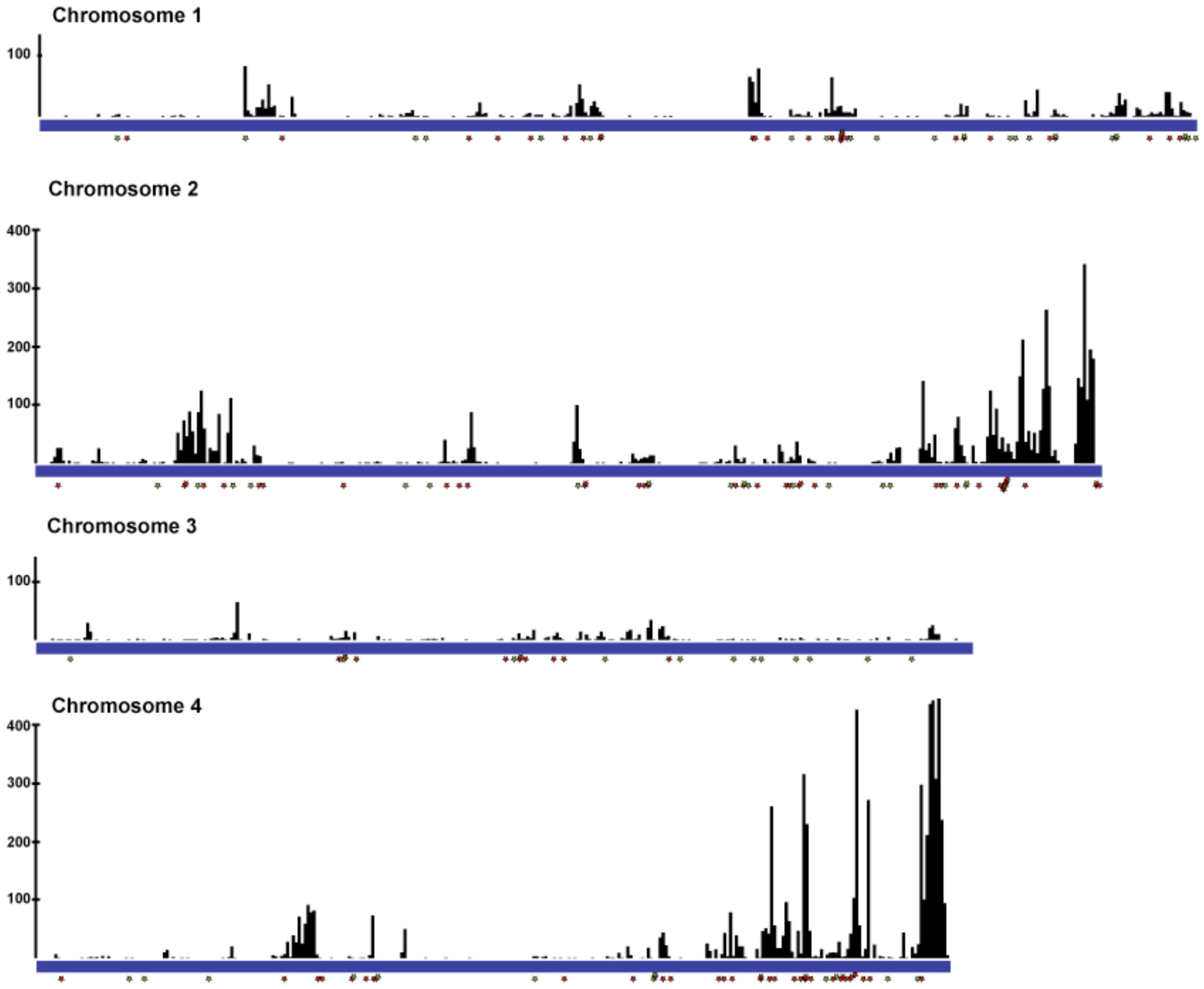

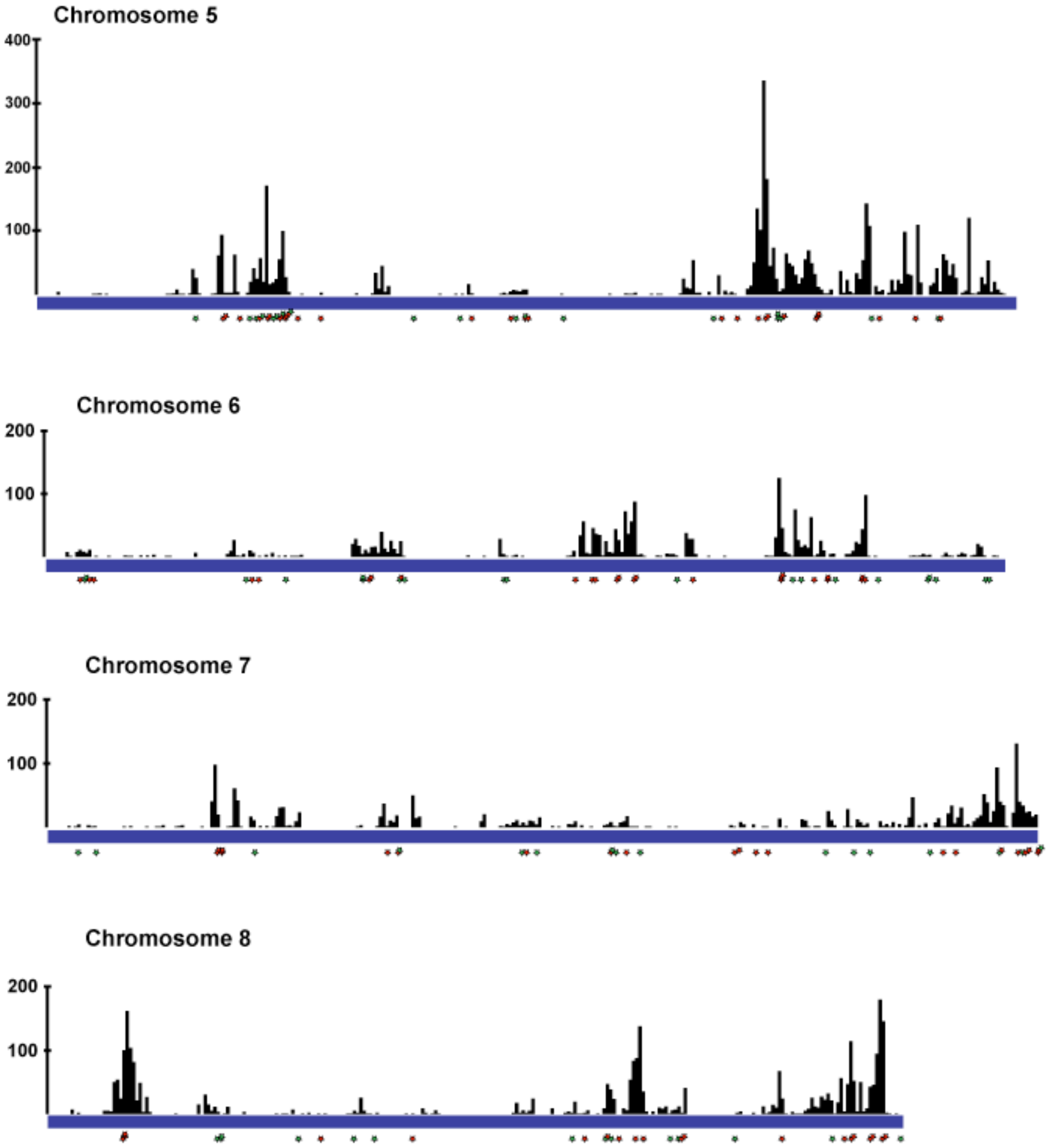
Chromosome 5
400
300
200
100
Chromosome 6
200
100
Chromosome 7
200
100
Chromosome 8
200
100

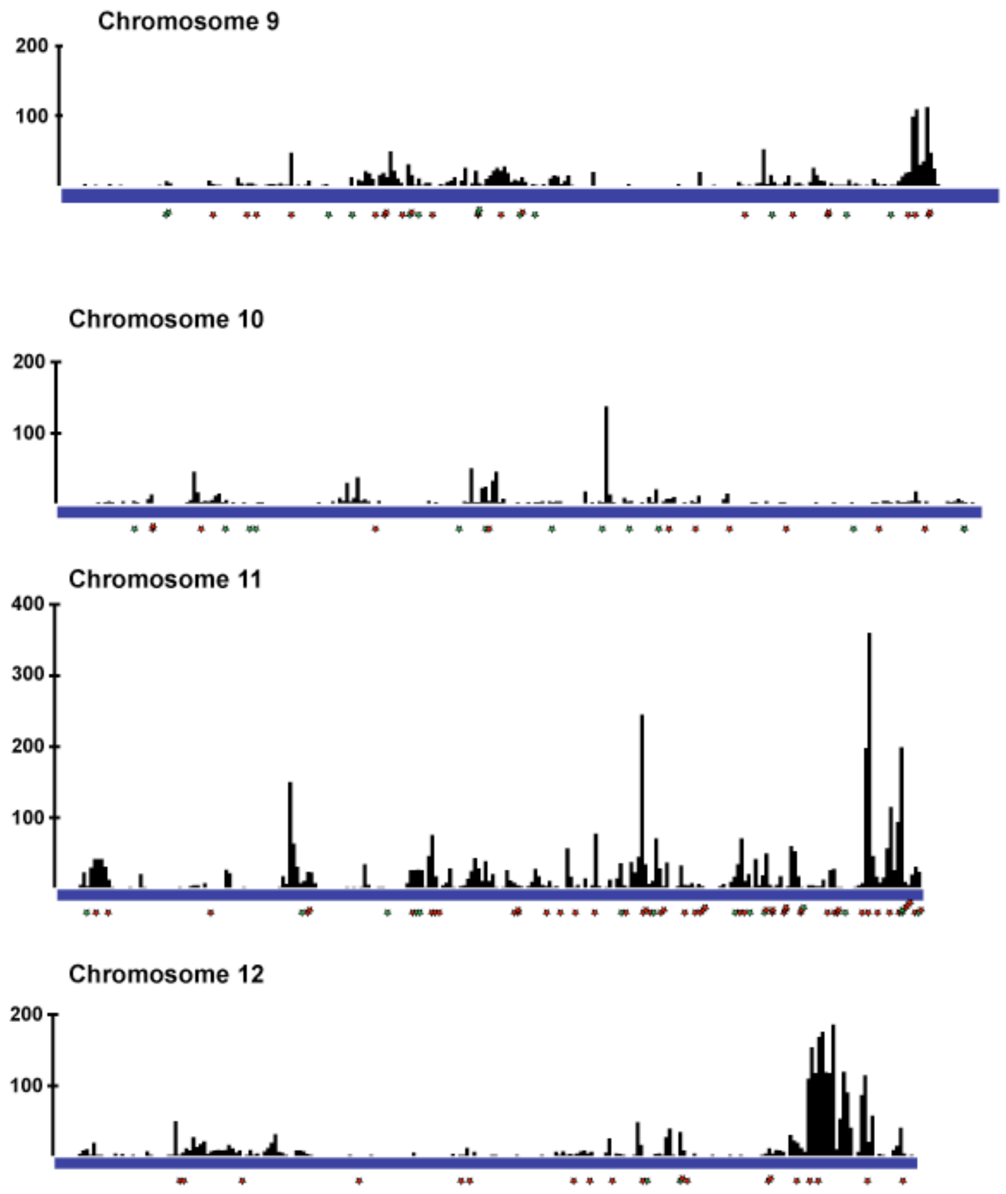
Chromosome 9
200
100
Chromosome 10
200
100
Chromosome 11
400
300
200
100
Chromosome 12
200
100

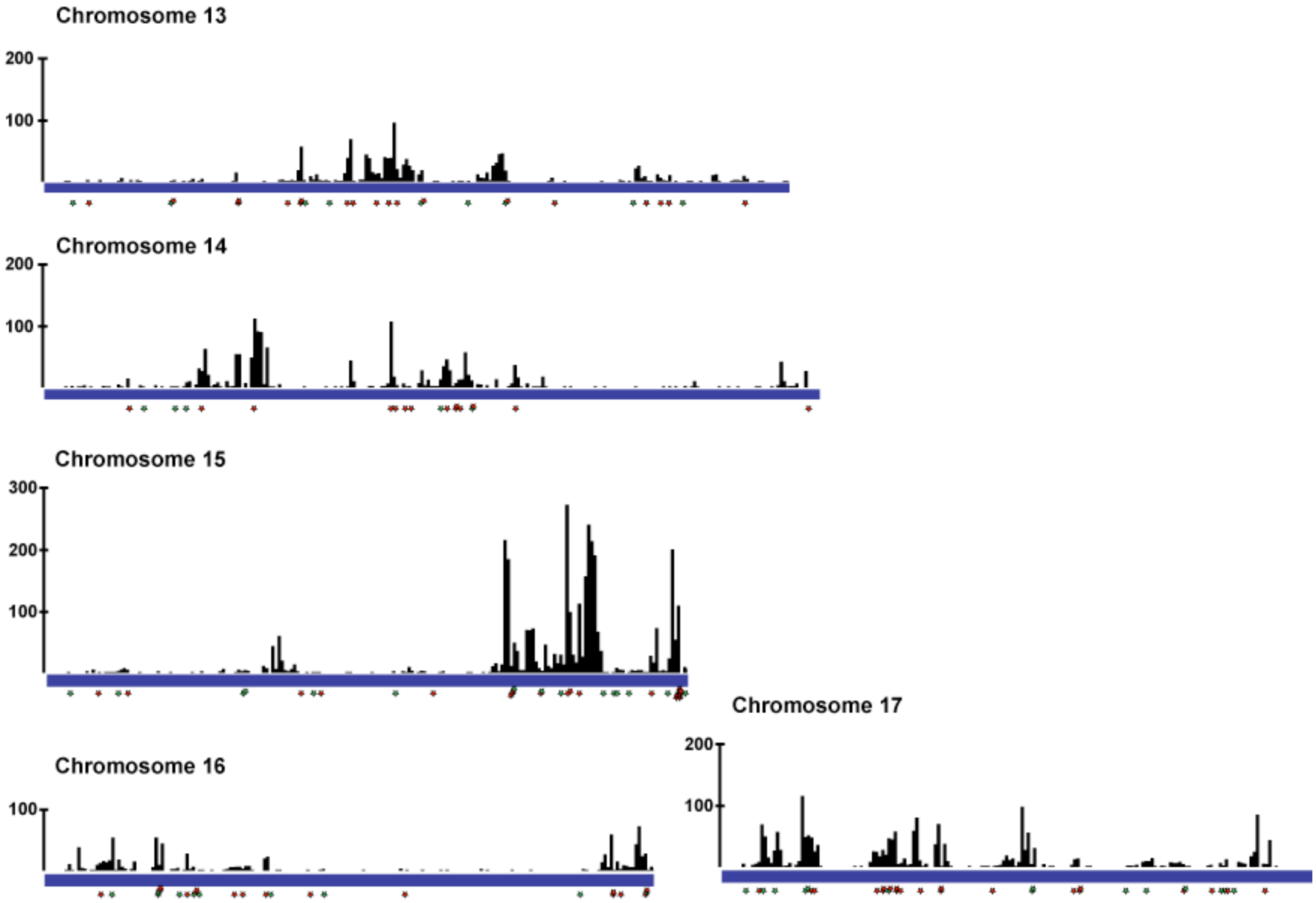
Chromosome 13
200
100
Chromosome 14
200
100
Chromosome 15
300
200
100
Chromosome 16
100
Chromosome 17
200
100

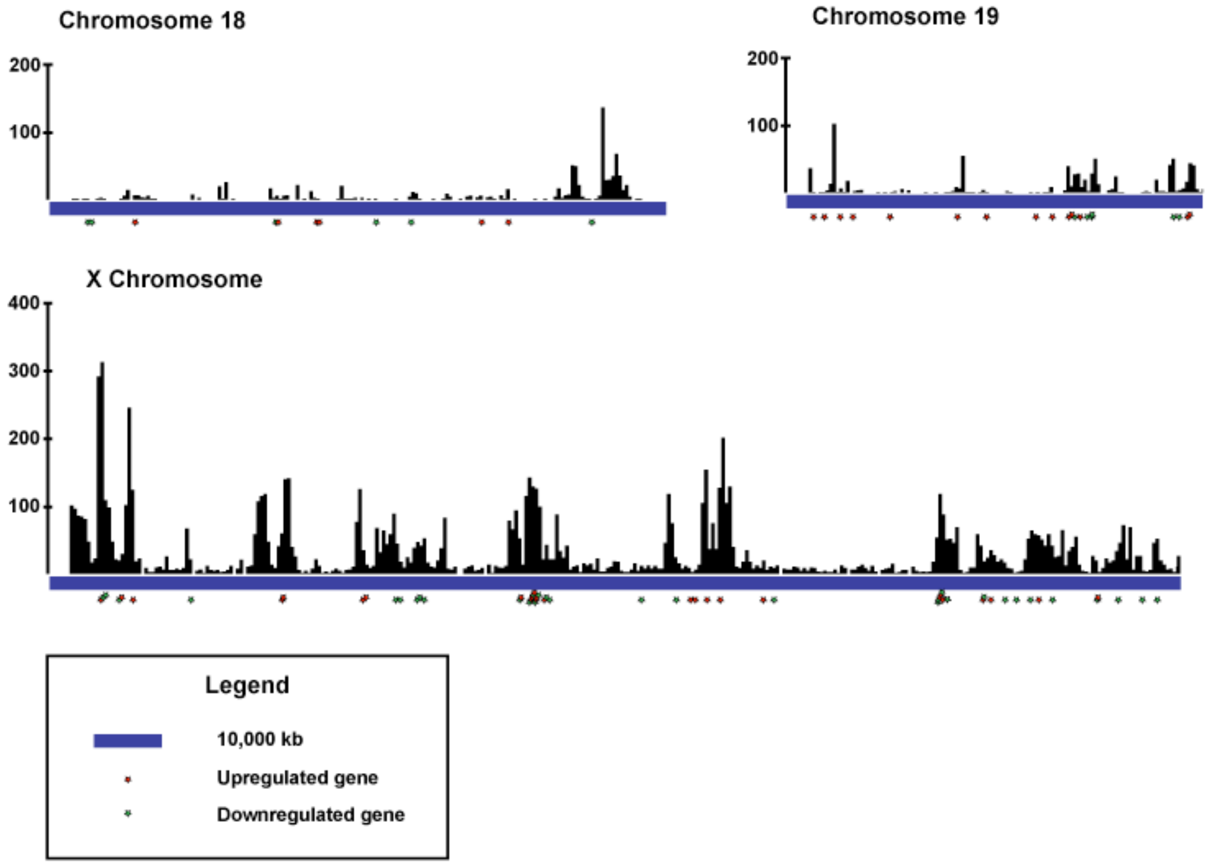
Chromosome 18
200
100
Chromosome 19
200
100
X Chromosome
400
300
200
100
Legend
10,000 kb
Upregulated gene
Downregulated gene
